# Utilizing deep learning via the 3D U-net neural network for the delineation of brain stroke lesions in MRI image

**DOI:** 10.1038/s41598-023-47107-7

**Published:** 2023-11-13

**Authors:** Parisa Soleimani, Navid Farezi

**Affiliations:** 1 Faculty of Physics, University of Tabriz, Tabriz, Iran; 2 Department of Engineering Sciences, Faculty of Advanced Technologies, University of Mohaghegh Ardabili, Namin, Iran

**Keywords:** Biological techniques, Neuroscience

## Abstract

The segmentation of acute stroke lesions plays a vital role in healthcare by assisting doctors in making prompt and well-informed treatment choices. Although Magnetic Resonance Imaging (MRI) is a time-intensive procedure, it produces high-fidelity images widely regarded as the most reliable diagnostic tool available. Employing deep learning techniques for automated stroke lesion segmentation can offer valuable insights into the precise location and extent of affected tissue, enabling medical professionals to effectively evaluate treatment risks and make informed assessments. In this research, a deep learning approach is introduced for segmenting acute and sub-acute stroke lesions from MRI images. To enhance feature learning through brain hemisphere symmetry, pre-processing techniques are applied to the data. To tackle the class imbalance challenge, we employed a strategy of using small patches with balanced sampling during training, along with a dynamically weighted loss function that incorporates f1-score and IOU-score (Intersection over Union). Furthermore, the 3D U-Net architecture is used to generate predictions for complete patches, employing a high degree of overlap between patches to minimize the requirement for subsequent post-processing steps. The 3D U-Net model, utilizing ResnetV2 as the pre-trained encoder for IOU-score and Seresnext101 for f1-score, stands as the leading state-of-the-art (SOTA) model for segmentation tasks. However, recent research has introduced a novel model that surpasses these metrics and demonstrates superior performance compared to other backbone architectures. The f1-score and IOU-score were computed for various backbones, with Seresnext101 achieving the highest f1-score and ResnetV2 performing the highest IOU-score. These calculations were conducted using a threshold value of 0.5. This research proposes a valuable model based on transfer learning for the classification of brain diseases in MRI scans. The achieved f1-score using the recommended classifiers demonstrates the effectiveness of the approach employed in this study. The findings indicate that Seresnext101 attains the highest f1-score of 0.94226, while ResnetV2 achieves the best IOU-score of 0.88342, making it the preferred architecture for segmentation methods. Furthermore, the study presents experimental results of the 3D U-Net model applied to brain stroke lesion segmentation, suggesting prospects for researchers interested in segmenting brain strokes and enhancing 3D U-Net models.

## Introduction

Stroke has been identified by the World Health Organization (WHO) as the third leading cause of death and disability worldwide^1–3^. The timely initiation of treatment has a significant impact on treatment outcomes^4^. Stroke lesions can be divided into two distinct parts: the infarct core, consisting of irreversibly damaged tissue, and the penumbra, comprising at-risk tissue that can still be salvaged if blood flow is restored^5^. The clinical identification and quantification of acute cores or penumbras are of utmost importance as they provide valuable insights into the potential extent of tissue that can be salvaged through alternative therapies^6^. Consequently, neuroimaging studies play a critical role in addressing fundamental inquiries about the functioning of the nervous system and the brain. Furthermore, they are essential for understanding the structural or functional alterations associated with various neurological disorders or brain lesions^7^.

Biomedical imaging plays a crucial role in diagnosing, prognosing, and treating a wide range of diseases, providing vital information^8^. When assessing brain function, magnetic resonance imaging (MRI) is the preferred imaging modality^9–11^. Due to its multi-spectral properties, MRI is favored by radiologists for diagnosing brain diseases, offering a superior means of detecting and evaluating potentially salvageable tissues. The advancements in MRI technology have significantly contributed to our understanding of brain structure and function^12–14^. Consequently, MRI is frequently employed to identify abnormalities in the posterior fossa, spinal cord, and other anatomical brain structures. Furthermore, MRI images are less susceptible to artifacts, providing an added advantage^15^. Researchers are utilizing various imaging techniques in conjunction with artificial intelligence (AI) to study the brain, aiming to improve patient outcomes and streamline the time-consuming processes involved in detecting and segmenting brain anomalies, as well as interpreting and analyzing complex brain imaging data^16,17^.

Over the past two decades, numerous deep learning (DL) neural network models, including convolutional neural networks (CNNs), have been developed and extensively utilized in classification applications to efficiently detect and segment organs and tissues, such as brain lesions, surpassing conventional methods^11,18,19^. Among these models, the 3D U-Net is a widely used deep learning architecture specifically designed for volumetric image segmentation tasks, particularly in medical imaging^3,20^. It's an extension of the U-Net and comprises an encoder-decoder structure with skip connections. The encoder reduces the spatial dimensions and increases feature channels, while the decoder upsamples the features and merges them with skip connections. This helps preserve spatial info for accurate segmentation. It excels in medical image tasks, such as brain tumor, organ, and vascular segmentation, due to its ability to handle volumetric data and capture spatial context effectively.

In this article introduces a novel, deep fully convolutional neural network model designed for segmenting stroke lesions using MRI images. The proposed model employs a deep learning algorithm to focus on decrypting the lesion zone. Currently, state-of-the-art (SOTA) networks for dense segmentation utilize asymmetric encoder-decoder architectures with short and long residual links^15^. Additionally, the incorporation of symmetrical modality extension allows for the extraction of more reliable image characteristics by leveraging symmetry between the brain's hemispheres. The suggested technique primarily utilizes a fully convolutional neural network for semantic image segmentation. This tool can be integrated into treatment decision workflows to quickly estimate the locations of lesion cores. The encoder component extracts features at various spatial resolutions through skip connections, which the decoder component uses to generate precise segmentation masks. Through concatenation, the decoder blocks are effectively combined with the skip connections.

## Methods and materials

### Dataset and data processing

**Figure 1:**
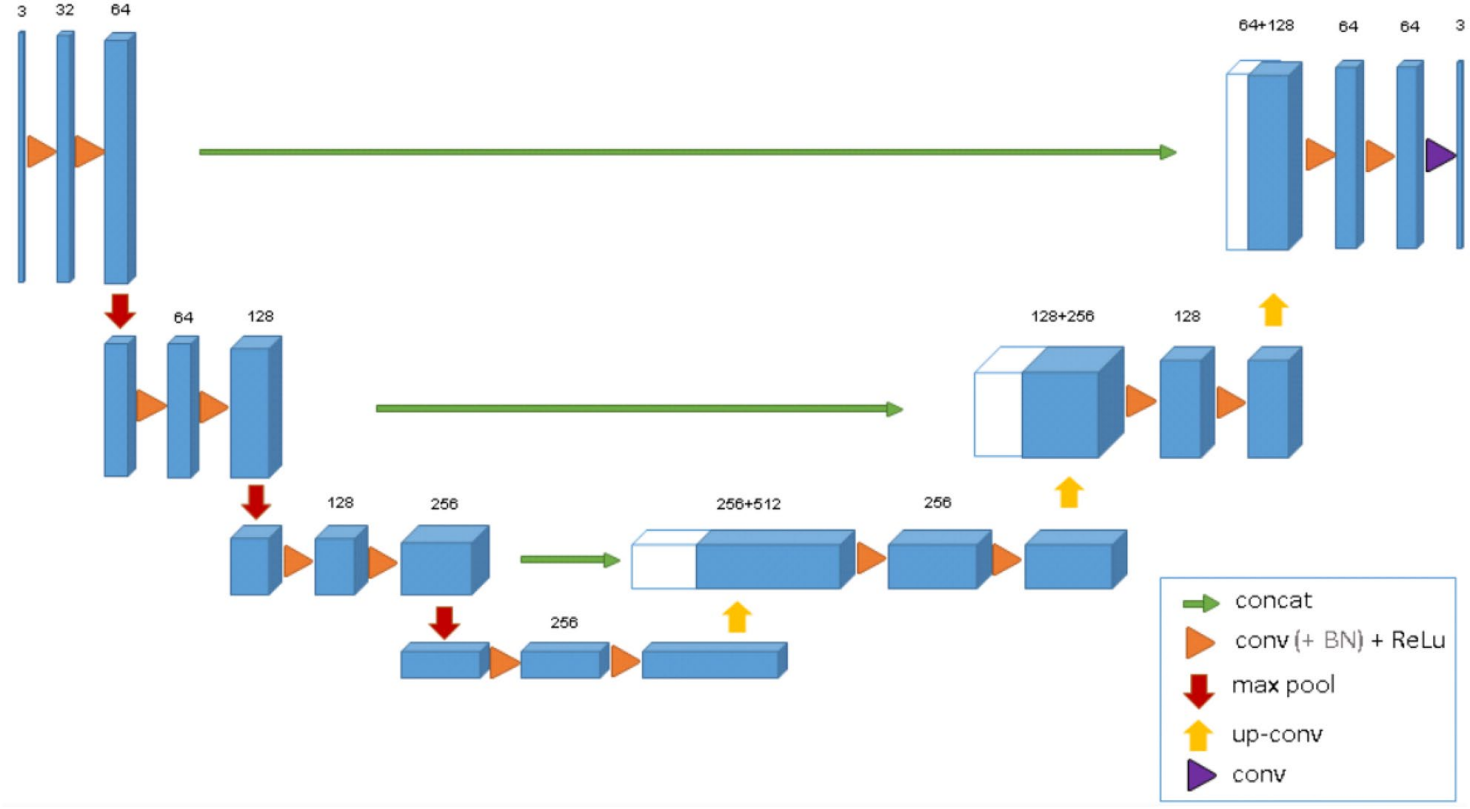
The 3D U-Net architecture consists of convolutional encoding and decoding units that take an image as input and create a segmentation feature map at each pixel class.

**Figure 2:**
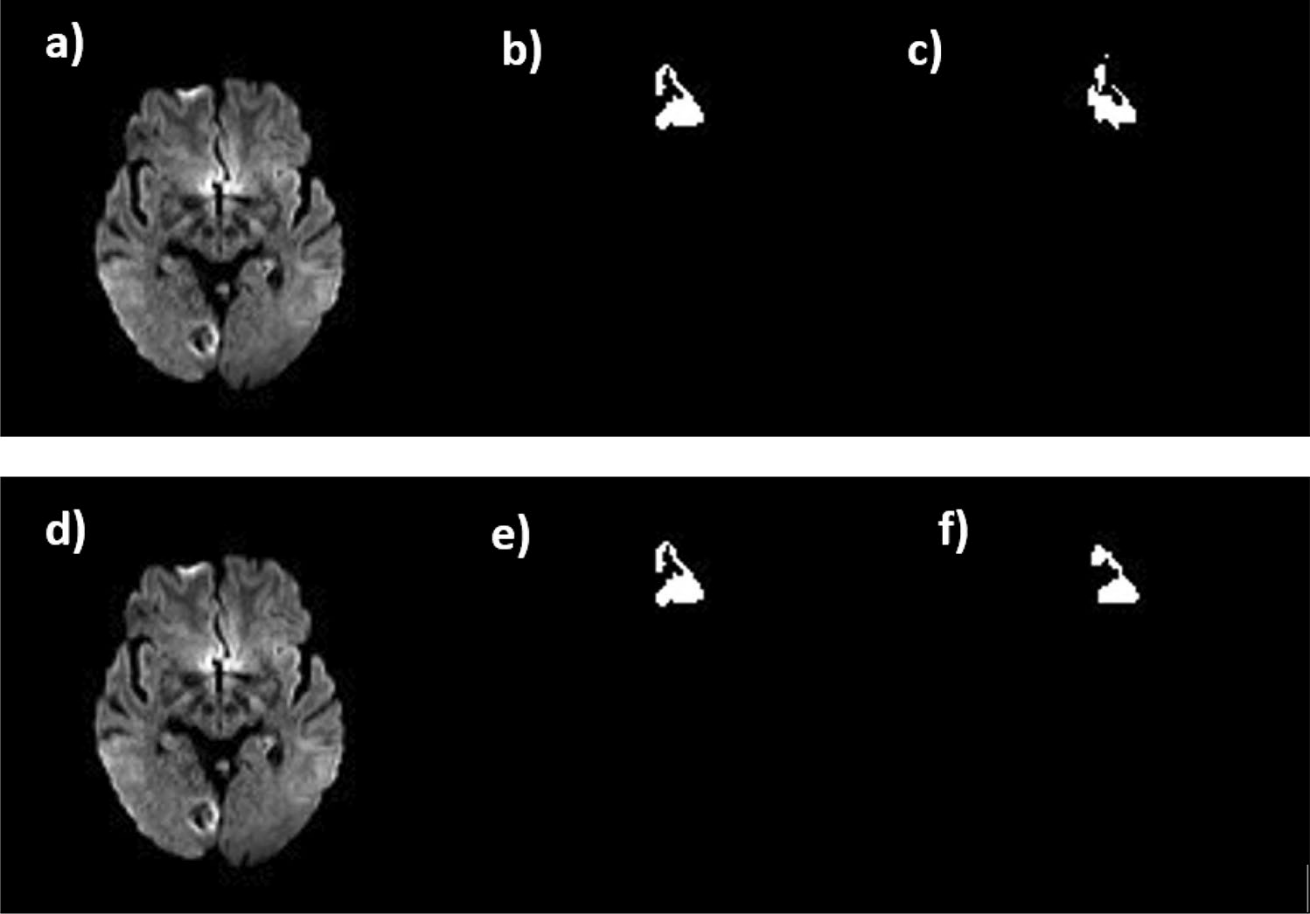
Extracted Results from U-Net (**a**,**d**) The MRI image of the brain, (**b**,**e**) image is either the Ground Truth (**c**,**f**) it is related that the power of estimating the lesion from the label which is (**a**–**c**) are for ResnetV2 and (**d**–**f**) are for seresnext101.

In this study, we utilized the dataset from the Sub-Acute Ischemic Stroke Lesion Segmentation (SISS) challenge, which is a subset of the larger Ischemic Stroke Lesion Segmentation (ISLES) dataset^21^. This publicly available dataset comprises 24 patients, each with a collection of 250 original images and corresponding lesion images. Out of these 24 patients, 20 were randomly selected for training, providing a substantial volume of data. The Flair modality was employed for our analysis in this specific research. The original dataset consisted of MRI scans, where the 3D segmentation volumes had dimensions of 256 × 256 × 32 with a resolution of 1 × 1 × 1 mm. To implement a segmentation algorithm, we decided to preprocess the original data for our deep learning architecture, as depicted in Fig. 1. Furthermore, all 3D MRIs were transformed into 2D image segments along the axial direction, as shown in Fig. 2. All 2D images were automatically refined to eliminate uninformative images lacking valuable data. This same process was applied to the ground truth data for stroke lesions. To evaluate the predictive accuracy of the model, we utilized the training data. Some patient slices had no available data, particularly at the upper and lower ends of the MRI segmentation. Consequently, these data-absent slices were excluded. We also resized the input images for ease of handling during upsampling and downsampling, removing unnecessary zero values. The dimensions of the input images now measure 64 × 128 × 3. For training and validation, custom data generators were established, using a batch size of 32 and an image input size of 64 × 128. This specific choice of batch size and input size was influenced by limitations in CPU memory. During the training phase, two distinct loss functions were integrated: binary cross-entropy (BCE) and Dice loss^22,23^. The combination of these two loss functions resulted in more distinct boundaries and superior performance compared to using each loss function independently.

The model was trained for a total of 250 epochs, utilizing a pre-existing encoder. However, it was observed that the model began to exhibit signs of overfitting after surpassing the 250-epoch milestone. Notably, the F1-score in the Seresnext101 model and the IOU-score in the ResnetV2 model showed substantial improvements in their respective performance metrics.

Within medical images, contrast-limited adaptive histogram equalization (CLAHE) has demonstrated favorable outcomes. This technique relies on partitioning the image into multiple non-overlapping regions of roughly equivalent sizes^20^. The approach aims to enhance specific portions of the image while avoiding undue impact on areas with distinct contrast. The image undergoes improvement through segmentation into two or more non-overlapping segments, followed by separate equalization, and eventual reintegration using interpolation methods^24^. Therefore, before inputting data into the network, CLAHE was applied to all input images. Figure 2 illustrates a brain image featuring a stroke lesion, both before and after CLAHE application, alongside the corresponding ground truth.

Various methods for augmenting the 2D stroke images have been employed. These methods include rescaling, horizontal flipping, rotation range, shear range, and zoom range, all aimed at generating a broader array of brain images. This augmentation process proves highly advantageous for enhancing the validation accuracy of the neural network. Following preprocessing, the data is shuffled, with 80 percent allocated for training and the remaining 20 percent reserved for validation.

### Implementation details

Network training and testing were conducted using an Intel Core i7 CPU operating at 2.60 GHz, coupled with an NVIDIA GeForce GTX 3060 Ti GPU featuring 16 GB of RAM, alongside a system memory of 32 GB.

### Standard encoder- decoder network architecture U-net

Derived from the conventional convolutional neural network, U-Net emerged in 2022 and was initially tailored for processing biomedical images, as depicted in Fig. 1. While a typical convolutional neural network is geared towards image classification, taking an image as input and producing a singular label as output, the demands of biomedical contexts entail not only detecting the presence of a condition but also pinpointing the affected region. The 3D U-Net was developed to address this challenge. It accomplishes this by performing pixel-wise classification, aligning the input and output dimensions. At first glance, its structure resembles the letter "U." The design exhibits symmetry, comprising two primary segments: the left portion, referred to as the contracting path, utilizes standard convolutional procedures, while the right portion, the expansive path, employs transposed 2D convolutional layers. It's noteworthy that each process consists of a pair of convolutional layers, and the channel count evolves from 1 to 32 due to the deepening convolution process. The downward-pointing red arrow signifies the max-pooling process, which reduces the image size by half. This sequence of operations is reiterated thrice more, leading to convergence at the lowest point of the architecture.

### Expansive path

Within the expansive path, the objective is to restore the image to its initial dimensions. Employing transposed convolution, an upsampling technique, enlarges the image size by applying padding to the original image and executing a convolution operation. Following the transposed convolution, the image undergoes expansion from 32 × 32 × 1024 to 64 × 64 × 512. Later, this resized image is fused with its corresponding image from the contracting path, yielding an amalgamated image measuring 64 × 64 × 1024. The underlying rationale is to amalgamate insights from prior layers, enhancing the precision of predictions.

Two additional convolutional layers are introduced. As before, this sequence is iterated three more times. Consequently, the architecture reaches its apex, where the ultimate step involves reshaping the image to meet our prediction criteria. The terminal layer constitutes a convolutional layer featuring a sole filter with dimensions of 1 × 1. It's worth noting the absence of dense layers throughout the entire network.

Following the U-net framework guidelines, a thorough evaluation of various backbones used in the network's encoder section was conducted. The attributes and performance metrics stemming from this evaluation are outlined in Table 1. Furthermore, the network proposed in this study underwent training for 250 epochs, employing a batch size of 32, a learning rate of 0.0001, and a threshold set at 0.5. This proposed network encompasses ResnetV2, ResNeXt, and others, each of which is detailed in Table 1 and Rows 1 through 5.

**Table 1:** Summary of performance of U-Net Architecture encoders.

Architecture	Summary of performance	Year of publication
VGG net	Transforming convolutions with substantial dimensions into 3 × 3 convolutions (amplifying depth, diminishing parameters, heightening accuracy)	2014
ResNet V2	Incorporating shortcut connections within grid blocks (enabling greater depth potential; improved gradient flow post propagation)	2015
ResNext	Establishing parallel pathways within the rosette block (enhancing precision)	2017
SeresNet	Utilizes squeeze-and-excitation blocks to empower the network with the capability for adaptive recalibration of features per-channel basis	2018
Seresnext101	Applies squeeze-and-excitation blocks to assist the network in dynamically readjusting features at the level of individual channels	2022

### Optimizer

The neural network's optimizer is an algorithm employed to modify the network's weights and biases, aiming to minimize a loss function. The primary objective of optimization is to find the specific weights and biases configuration that results in the least loss. Various optimization approaches exist, including gradient descent, stochastic gradient descent, Adam, RMSprop, and Adagrad. To expedite convergence and optimize memory utilization, the Adam optimizer was used with a learning rate of 0.0001^25^.

### Evaluation metrics

#### Dice loss

The Dice loss serves as a loss function used within neural networks to quantify the resemblance between two sets of data. It finds widespread application in image segmentation assignments, where the objective is to categorize individual pixels in an image into specific classes. This coefficient assesses the degree of overlap between the two data sets, and the Dice loss is essentially the negation of this coefficient. Utilizing the Dice loss as a metric allows for assessing a neural network model's effectiveness in handling image segmentation tasks. The formulation is provided below^26–28^:

1 $$2\times \frac{\left|A\cap B\right|}{\left|A\right|+\left|B\right|},$$

where A and B are sets of data points.

#### IOU

The Intersection over Union (IOU) quantifies the degree of overlap between two samples, and its formulation is as follows^29^:

2 $$\frac{\left|A\cap B\right|}{\left|A\cup B\right|},$$

where A and B are sets of data points.

${f}_{1}$-score: The f1-score is a measure of the accuracy of a model's predictions and is defined by an equation.

3 $$2\times \frac{Precision\times Recall}{Precision+Recall}.$$

Precision is the fraction of true positives out of all predicted positives, and recall is the fraction of true positives out of all actual positives^10,30^.

## Results

### Output comparison

**Figure 3:**
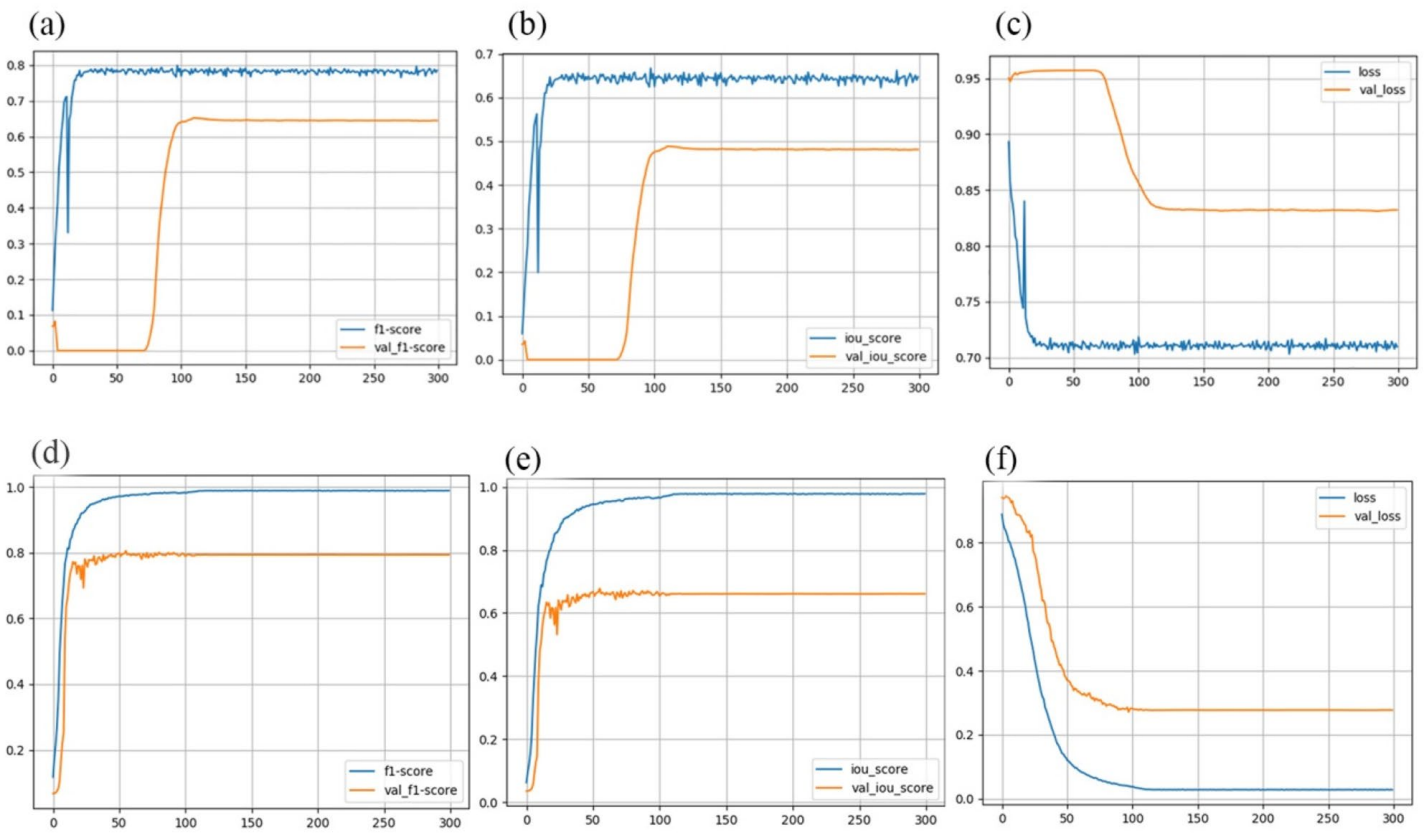
Confusion matrix for the test and train data of the (**a**) f1-score, (**b**) IOU-score and (**c**) loss for ResnetV2 and (**d**) ${f}_{1}$-score, (**e**) Iou-score and (**f**) loss for seresnext101 curve versus epoch.

The U-Net with ResnetV2 serving as the pre-trained encoder is widely recognized as the state-of-the-art (SOTA) model for segmentation tasks. For the IOU-score, ResnetV2 is employed, while Seresnext101 is used for the f1-score. The newly proposed model showcased superior performance across all three metrics, as detailed in Table 2, underscoring its superiority over the other considered backbones. The f1-score and IOU-score metrics were computed across various backbones. From Table 2, it is evident that the best f1-score is attributed to Seresnext101, whereas the finest IOU-score is attributed to ResnetV2. The threshold value was set at 0.5. The evaluation of the confusion matrix data for both the test and train data of the ResnetV2 model, depicted in Fig. 2, highlights the IOU-score, loss, and f1-score trends across epochs for the model. These outcomes, presented in Fig. 3 and Table 2, summarize the achieved results.

**Table 2:** The scores obtained from the evaluation result for the deep ML-based pre-trained models.

Model-name	Trainable params	IOU-score	${\mathrm{f}}_{1}-\mathrm{score}$	weights	Loss	Params(m)
Vgg	26,578,622	0.76034	0.78502	Imagenet	0.40569	13 M
ResnetV2	17,526,487	0.88342	0.64869	Imagenet	0.87562	9 M
Seresnet18	19,542,453	0.59249	0.7456	Imagenet	0.73456	22 M
Resnext	33,215,492	0.62813	0.86954	Imagenet	0.80664	21 M
Seresnext101	37,546,285	0.66105	0.94226	Imagenet	0.32654	19 M

For each model, a set of 6 random images was selected from the testing dataset. In each set, the image on the left displays the brain's MRI scan, while the middle image represents either the Ground Truth or the lesion identified by the physician. The image on the right is linked to the ability to predict the lesion based on the predefined label.

## Discussion

The objective of this study is to evaluate various backbone architectures within the U-Net neural network framework for deep learning-based segmentation. Specifically, the study focuses on VGG, ResnetV2, Seresnet18, Resnext, and Seresnext101 backbones in their capability to predict stroke lesion diagnoses. The research utilized the Ischemic Stroke Lesions Segmentation Challenge (SISS) dataset, a subset of the Ischemic Stroke Lesions Segmentation (ISLES) dataset. This publicly accessible dataset comprises images from 24 patients, with each patient having 153 original images and images of lesions. Among these patients, 20 were randomly allocated for training, while the remaining four were designated for testing. Consequently, a substantial volume of data was generated. The SISS challenge provides access to 32 training cases from the dataset, which encompass four different MRI methods along with their corresponding ground truth annotations. For this study, the Flair modality was employed.

Among the various deep learning segmentation methods (VGG, ResnetV2, Seresnet18, Resnext50, and Seresnext101), ResnetV2 exhibited the most proficient performance in subject segmentation based on IOU outcomes, while Seresnext101 demonstrated superior segmentation outcomes based on the F1-score. The attained F1-score accuracies were 0.94226 and 0.88342, respectively.

Recent attention has been directed towards MRI-based medical image processing for brain segmentation research, driven by the growing need to efficiently and impartially analyze large volumes of medical data. Given the high mortality rate associated with brain diseases, early detection and intervention are crucial. However, the intricate nature of brain tissue makes manual diagnosis time-consuming and reliant on operators. Therefore, this study introduces a valuable model built on transfer learning to classify brain disorders in MRI scans. The achieved F1-scores using the proposed classifier substantiate the effectiveness of the approach presented in this investigation. Notably, Seresnext101 yielded the highest F1-score of 0.94226, and ResnetV2 recorded the top IOU-score of 0.88342. These results establish Seresnext101 and ResnetV2 as the most optimal architectures for segmentation methods.

## Conclusion and future work

In conclusion and as a bridge to future work, this research embarked on the contemporary approach of utilizing the 3D U-Net model for predicting stroke lesions, serving as a modern diagnostic method. The core objective of this study was to identify the most effective predictive models for various diseases, with a particular focus on the prognosis of stroke lesions—a condition characterized by diverse definitions and causes. Our current exploration encompassed the evaluation of both traditional models and deep learning techniques for well-known diseases within our dataset, with the aim of distinguishing and comparing their predictive capabilities. In future, it intend to build upon these findings by addressing the following aspects, Expanding datasets to include a larger and more diverse set of medical images to enhance the generalizability of models. Implementing quality control measures to ensure consistent and accurate annotations, mitigating potential data quality and annotator variability issues. Exploring interpretability techniques for our deep learning models to make their predictions more transparent and clinically interpretable. Optimizing our models for computational efficiency to increase accessibility across various healthcare settings. By embracing these future directions, we aim to continue advancing the field of medical image analysis, contribute to more effective diagnostic methods, and ultimately improve patient outcomes. This research lays the foundation for an ongoing commitment to enhancing the practicality and effectiveness of our proposed methodology.

## Data Availability

Data are available from the corresponding author on request (https://zenodo.org/record/7960856#.ZK5or-xBzmE).

## References

[1] Siuly S, Zhang Y (2016). Medical big data: neurological diseases diagnosis through medical data analysis. Data Sci. Eng..

[2] Raghavendra U, Acharya UR, Adeli H (2019). Artificial intelligence techniques for automated diagnosis of neurological disorders. Eur. Neurol..

[3] Clèrigues A (2019). Acute ischemic stroke lesion core segmentation in CT perfusion images using fully convolutional neural networks. Comput. Biol. Med..

[4] Wilson JE (2020). Delirium. Nat. Rev. Dis. Primers.

[5] Alaya IB (2022). Applications of artificial intelligence for DWI and PWI data processing in acute ischemic stroke: Current practices and future directions. Clin. Imaging.

[6] Goyal M (2022). How can imaging in acute ischemic stroke help us to understand tissue fate in the era of endovascular treatment and cerebroprotection?. Neuroradiology.

[7] N. Kinany, E. Pirondini et al. Spinal Cord fMRI: A New Window into the Central Nervous System. Neuroscientist. 10738584221101827, 2022. 10.1177/10738584221101827 PMC10623605 35822665

[8] Kumar Y, Koul A (2022). Artificial intelligence in disease diagnosis: A systematic literature review, synthesizing framework and future research agenda. J. Ambient Intell. Humaniz. Comput..

[9] Kaur N, Soumya SS, Mazumder N (2022). Advanced Magnetic Resonance Imaging (MRI) of Brain. Advances in Brain Imaging Techniques.

[10] Pinto A (2018). Stroke lesion outcome prediction based on MRI imaging combined with clinical information. Front. Neurol..

[11] Moeskops P (2018). Evaluation of a deep learning approach for the segmentation of brain tissues and white matter hyperintensities of presumed vascular origin in MRI. NeuroImage Clin..

[12] Majib MS (2021). Vgg-scnet: A vgg net-based deep learning framework for brain tumor detection on mri images. IEEE Access.

[13] Guan Y (2021). A framework for efficient brain tumor classification using MRI images. Math. Biosci. Eng.

[14] M. A. Hafeez and et al, "Brain Tumor Classification Using MRI Images and Convolutional Neural Networks," In 2022 30th Signal Processing and Communications Applications Conference (SIU), pp. 1–4, 2022.

[15] D’Arco F (2022). Guidelines for magnetic resonance imaging in pediatric head and neck pathologies: A multicentre international consensus paper. Neuroradiology.

[16] Meshaka R (2022). Artificial intelligence applied to fetal MRI: A scoping review of current research. Br. J. Radiol..

[17] Bonkhoff AK (2022). Precision medicine in stroke: Towards personalized outcome predictions using artificial intelligence. Brain.

[18] Khan MS (2022). Accurate brain tumor detection using deep convolutional neural network. Computat. Struct. Biotechnol. J..

[19] Zhang N (2011). Kernel feature selection to fuse multi-spectral MRI images for brain tumor segmentation. Comput. Vision Image Understand..

[20] Ronneberger O, Navab N (2015). U-net: Convolutional networks for biomedical image segmentation. International Conference on Medical image computing and Computer-Assisted Intervention.

[21] [online] https://isles22.grand-challenge.org/dataset/.

[22] Zunair H, Ben HA (2021). Sharp U-Net: Depthwise convolutional network for biomedical image segmentation. Comput. Biol. Med..

[23] Hara K, Hara K (2015). Analysis of function of rectified linear unit used in deep learning. 2015 International Joint Conference on Neural Networks (IJCNN).

[24] Alom MZ (2019). Recurrent residual U-Net for medical image segmentation. J. Med. Imaging.

[25] Bae MH, Pan R, Wu T, Badea A (2009). Automated segmentation of mouse brain images using extended MRF. Neuroimage.

[26] O’Leary-Roseberry T (2022). Learning high-dimensional parametric maps via reduced basis adaptive residual networks. Comput. Methods Appl. Mech. Eng..

[27] Ingle A (2022). Efficient segmentation and classification of the tumor using improved encoder-decoder architecture in brain MRI images. Int. J. Electr. Comput. Eng. Syst..

[28] Linqi J (2022). Glioma classification framework based on SE-ResNeXt network and its optimization. IET Image Process..

[29] Yalçın S (2022). Brain stroke classification and segmentation using encoder-decoder based deep convolutional neural networks. Comput. Biol. Med..

[30] Kaur N, Sahoo SS, Rana SS, Kaur N (2022). Advanced magnetic resonance imaging (MRI) of brain. Advances in Brain Imaging Techniques.

